# Mechanism of Huangqi–Honghua combination regulating the gut microbiota to affect bile acid metabolism towards preventing cerebral ischaemia–reperfusion injury in rats

**DOI:** 10.1080/13880209.2022.2136209

**Published:** 2022-10-28

**Authors:** Kai Wang, Yue Chen, Jinyi Cao, Ruimin Liang, Yi Qiao, Likun Ding, Xiaojuan Yang, Zhifu Yang

**Affiliations:** a Department of Pharmacy, Xijing Hospital, Fourth Military Medical University, Xi’an, China; b Pharmacy Department, Xi’an Daxing Hospital, Xi’an, China

**Keywords:** Traditional Chinese Medicine, ischaemic stroke, inflammation

## Abstract

**Context:**

Effective treatment of ischaemic stroke is required to combat its high prevalence and incidence. Although the combination of *Astragalus membranaeus* (Fisch.) Bge. (Fabaceae) and *Carthamus tinctorius* L. (Asteraceae) is used in traditional Chinese medicine for the treatment of stroke, its underlying mechanism remains unclear.

**Objective:**

The objective of this study is to elucidate the mechanism underlying Huangqi-Honghua (HQ-HH) for the treatment of ischaemic stroke by gut microbiota analysis and metabonomics.

**Materials and methods:**

Sprague–Dawley rats were randomly assigned to the sham, model, HQ-HH, and Naoxintong (NXT) groups. The middle cerebral artery occlusion-reperfusion model was established after 7 days of intragastric administration in the HQ-HH (4.5 g/kg, qd) and NXT (1.0 g/kg, qd) groups. The neurological examination, infarct volume, gut microbiota, bile acids, and inflammation markers were assessed after 72 h of reperfusion.

**Results:**

Compared with the model group, HQ-HH significantly reduced the neurological deficit scores of the model rats (2.0 ± 0.2 vs. 3.16 ± 0.56), and reduced the cerebral infarct volume (27.83 ± 3.95 vs. 45.17 ± 2.75), and reduced the rate of necrotic neurons (26.35 ± 4.37 vs. 53.50 ± 9.61). HQ-HH regulating gut microbiota, activating the bile acid receptor FXR, maintaining the homeostasis of bile acid, reducing Th17 cells and increasing Treg cells in the rat brain, reducing the inflammatory response, and improving cerebral ischaemia–reperfusion injury.

**Conclusions:**

These data indicate that HQ-HH combination can improve ischaemic stroke by regulating the gut microbiota to affect bile acid metabolism, providing experimental evidence for the wide application of HQ-HH in clinical practice of ischaemic stroke.

## Introduction

Stroke is the second leading cause of death worldwide. Owing to the increase in the global ageing population, the incidence of stroke is also increasing. Many cases of stroke are caused due to an abrupt blockage of an artery, namely ischaemic stroke, which accounts for about 70–80% of all strokes (Arya and Hu 2018; Paul and Candelario-Jalil 2021). Therefore, the prevention and treatment of ischaemic stroke has become a major public health problem, and the development of ischaemic stroke drugs is required.

Gut microbiota is a complex community of microorganisms and plays a major role in health and disease (Adak and Khan 2019; Angelucci et al. 2019). Gut microbiota regulates the human digestive, metabolic, inflammatory, and immune functions (Cryan et al. 2019). A close relationship exists between gut microbiota and ischaemic stroke. Ischaemic stroke alters the composition of the intestinal microbiota, and intestinal microbiota can affect the occurrence and development of ischaemic stroke (Nam 2019). Alteration of gut microbiota can protect from cerebral ischaemia-induced brain injury. Intestinal flora can alter the immune homeostasis of the small intestine, which leads to an increase in Treg cells and a reduction in interleukin-17 (IL-17) positive γδT or T helper cells 17 (Th17) through altered dendritic cell activity, which increases IL-10 and reducing IL-17, thereby reducing systemic inflammation after acute ischaemic stroke and exerting neuroprotective effects (Benakis et al. 2016). Intestinal flora is involved in the synthesis, metabolism, and signal transduction of bile acids. Bile acids play an important role in regulating various pathophysiological processes such as lipid, glucose stability, inflammatory response, intestinal microflora structure and growth, and the occurrence and development of atherosclerosis, obesity, cholestasis, and other diseases (Li and Chiang 2014). Therefore, we determined the relationship between the intestinal flora and bile acid metabolism changes in ischaemic stroke from a multi-group approach, which is particularly important for understanding the effect of drug treatment.

Presently, the clinical treatment methods and drugs for ischaemic stroke (such as recirculation surgery or recombinant tissue plasminogen activator) have the problems of a narrow treatment time window and single mechanism of action, which cannot achieve satisfactory treatment effects in most patients. Traditional Chinese Medicine (TCM) is a product under the guidance of a holistic view of TCM and syndrome differentiation theory. It is advantageous for the treatment of complex diseases and has rich clinical experience in the prevention and treatment of ischaemic stroke. *Astragalus membranaeus* (Fisch.) Bge. (Fabaceae) and *Carthamus tinctorius* L. (Asteraceae), as TCM, have the characteristics of multi-component and multi-target, and are widely used in the treatment of cardiovascular and cerebrovascular diseases.

*Radix Astragali* (Huangqi, HQ), the dried roots of *A. membranaeus,* has been extensively used in TCM for patients with stroke or chronic debilitating diseases. The dried florets of *C. tinctorius* (Honghua, HH) have been extensively used in TCM to treat stroke and coronary heart disease (Yue et al. 2013). Huangqi and Honghua are the primary active constituents in the Buyang Huanwu decoction. They are a well-known TCM formula reported in “Yilin Gaicuo” written by Qingren Wang (Zhang H et al. 2010). Our previous study showed that HQ-HH (5:1) with cerebral infarction has a certain brain-protective effect (Cao et al. 2014; Chen et al. 2022). The main active ingredients of Honghua are Hydroxysafflor yellow A (HSYA) (Xue et al. 2021), and oral administration of HSYA can reduce obesity by regulating intestinal microflora and serum metabolism (Liu et al. 2018). *Astragalus* extract inhibits colon cancer in CT26-bearing mice by regulating gut microbiota imbalance and intestinal short-chain fatty acid content (Gu et al. 2021). However, it is not known whether the protective effect of HQ-HH on CI/RI is related to gut microbiota.

Based on the protective effect of HQ-HH on CI/RI in rats, 16S rRNA sequencing was performed to determine the composition of intestinal microflora. Liquid chromatography-mass spectrometry (LC/MS) was used to investigate bile acid metabolomics. We determined whether HQ-HH can regulate gut microbiota-mediated bile acid metabolism, regulate the immune balance of Th17 and regulatory T cells (Tregs cells), affect the expression of IL-17A and IL-10, and reduce inflammation after CI/RI to play a protective role in the brain.

## Materials and methods

### Materials

The extracts of HQ and HH (5:1 ratio) were obtained from Guangdong Yifang Pharmaceutical Co., Ltd. (batch number: 2020053). We examined the source, character, identification, and content of the extract. The quality standards were in accordance with the provisions of Chinese Pharmacopoeia. Naoxintong Capsule (Batch No.: 2002107212, Shaanxi Buchang Pharmaceutical Co., Ltd.).

### Animal study

Forty specific-pathogen-free (SPF)-grade Sprague–Dawley (SD) rats (male, 250 ± 30 g) were purchased from the Animal Experiment Centre of Fourth Military Medical University (Shaanxi, Xi’an; Animal Production Licence No. SCXK, Shaan-2020-001). The animal experiments performed in this study were approved by the Ethics Committee of Xijing Hospital, Fourth Military Medical University. After 1 week of adaptive feed, all 40 rats were randomly assigned to four groups: sham group, model group, HQ–HH group, and NXT group.

SD rats in the Sham operation group and the model group were administered with normal saline (10 mL/kg) once a day, those in the HQ-HH group were administered the HQ-HH combination (10 mL/kg) once a day, while those in the NXT group were administered Naoxintong capsule (10 mL/kg) once a day. The drug concentration of the administration groups was 0.45 g/mL for the HQ-HH group and 0.1 g/mL for the NXT group. The MCAO/R model was established after 7 days of continuous administration in the HH and NXT groups, followed by 3 days of continuous administration. All neurological examinations were performed after 72 h of cerebral ischaemia–reperfusion. The rats were anaesthetized via intraperitoneal injection of 3% sodium pentobarbital (Merck Company, USA). Then, the rats were sacrificed and their brain, small intestine, and faeces samples were collected; the brain tissues were then subjected to haematoxylin–eosin (Biyuntian Biotechnology Co., Ltd., Shanghai, China) staining analysis and fixed in 4% paraformaldehyde (White Shark Biological Technology Co., Ltd., Hefei, China). The tissue and faeces were stored at −80 °C for subsequent analyses.

### MCAO/R model establishment

The SD rats were anaesthetized with 3% sodium pentobarbital. The body temperature of rats was maintained at approximately 37 °C during the operation until anaesthesia. The CI/RI model was adapted from Longa et al. (1989) and Koizumi et al. (1986). The left common carotid artery (CCA), external carotid artery (ECA), and internal carotid artery (ICA) were separated by operation. The CI/RI model was established by inserting a nylon wire (approximately 1.8 cm) from the CCA to the ICA, avoiding the pterygopalatine artery. Laser Doppler Flowmetry was used to monitor cerebral blood flow. After inserting the nylon wire, the ICA was ligated to complete the ipsilateral ischaemia. After 2 h of ischaemia, the nylon wire was gently pulled out to form reperfusion. The wound was disinfected with iodine and then sutured.

### Evaluating the MCAO/R model

We employed Bederson’s scoring method to reflect the damage to the neurological functions (Bederson et al. 1986). Next, TTC staining was performed to reflect the percentage of cerebral infarction in the brain volume. The complete brain tissue sections were obtained from the rats immediately after the anaesthesia, excised into 2 mm thick sections, stained with 2% 2,3,5-triphenyl tetrazolium chloride (TTC, Sigma-Aldrich, USA) for 30 min, and fixed in 4% formaldehyde immersion for 24 h. We then used Image J 5.1 software to calculate the area of cerebral infarction. The percentage of cerebral infarction was calculated using the following formula: Cerebral infarction (%) = (infarct area × thickness)/the volume of the non-ischaemic hemisphere × 100%.

### Histopathological analyses

The brain tissue was fixed with 4% paraformaldehyde, embedded, sliced at 4 μm thickness, and stained with haematoxylin-eosin in accordance with the standard procedure. The pathological morphology of the brain tissue was analysed using the Image-Pro Plus 7.0 software (Media Cybernetics Inc., Bethesda, MD, USA).

### Immunofluorescence studies

Immunofluorescence staining revealed that 5% goat serum was incubated at room temperature for 1 h to block the non-specific binding. Antibodies against IL-17A (Abcam, UK, No. ab79056), Foxp3 (Abcam, No. Ab215206), CD4 (Abcam, No. (Ab237722), and IL-17 (Servicebio, China, No. GB11110-1) were used and incubated at 4 °C overnight. After the sections were washed, the goat anti-rabbit secondary antibody coupled with Alexa 488 was incubated at room temperature for 50 min. Fluorescently labelled slices were covered with Prolong-Gold antifade mounting medium (Unique, China). The negative controls were examined overnight with PBS and 0.1% Triton-X. Leica TCS SP5 confocal (Leica, Germany) laser scanning microscope was used to capture all fluorescence images, while the Caseviewer 2.0 was used to process all images.

### Gut microbiota analysis

All faeces for 16S rRNA sequencing were collected at a fixed time point (08:00 AM) through abdominal massage. All faecal samples were subjected to the same procedures for DNA extraction and PCR amplification by the same laboratory staff. In addition, all experimental operations were conducted in a sterile environment. DNA was extracted using the E.Z.N.A.^Ⓡ^ Stool DNA Kit (Omega Bio-Tek, Inc., GA, USA), while excluding the lysis steps, and stored at −20 °C for further analyses. The extracted DNA from each sample was used as the template to amplify the V3–V4 region of 16S rRNA genes. The quality and concentration of the DNA were tested using the Nanodrop ND-1000 Spectrophotometer (Thermo Electron Corporation, USA). The final PCR products were purified from unincorporated nucleotides and primers using the EasyCycler 96 PCR System (Analytik Jena Corp., AG). The purified samples were normalized to equal DNA concentration using the MiSeq platform (Illumina Inc., USA) and sequenced by Shanghai Mebio Biomedical Technology Co., Ltd.

### Western blotting

The small intestine and surrounding tissues of cerebral infarction were quickly removed. The rat intestines and cerebral peri-infarct tissue were homogenized in lysis buffer containing Tris–HCl (pH 6.8/8.8, Legian Biotechnology Co., Ltd., Beijing, China) and 2% sodium dodecyl sulphate (SDS, Biyuntian Technology Co., Ltd., Beijing, China). The protein was separated by 8% SDS-polyacrylamide gel and then transferred onto the polyvinylidene fluoride membrane (Milliepol, USA). After blocking with a 5% skim milk solution, the primary antibody was incubated overnight at 4 °C. The primary antibodies used in the study were as follows: ZO-1 (Cell Signalling Technology, USA, #5406), Occludin (Abcam, ab167161), Claudin-1 (Abcam, ab180158), FXR (Abcam), IL-17A (Abcam, ab193955), IL-10 (Abcam, ab9969), IL-6 (Abcam, ab9324), MMP-9 (Abcam, ab76003), or β-actin (Abcam, ab8226). After washing, the membranes were incubated with horseradish peroxidase-labelled goat anti-rabbit antibody or goat anti-mouse antibody (Cell Signalling Technology, USA). Western blotting detection reagent (Zeta LIFE, USA) was used to detect chemiluminescence for 2 h. Finally, the Image J software (NIH, Bethesda, MD, USA) was used to detect the intensity of the bands by chemiluminescence and normalized to the intensity of the β-actin bands.

### Metabolomics analyses

The stool specimens (10 mg) were added to 1000 μL of methanol (–20 °C). The samples were spun for 60 s and centrifuged at 12,000 rpm for 10 min at 4 °C. The supernatant was transferred into a 300 μL vial for LC/MS analysis.

### Statistics analysis and bioinformatics

Using the Kruskal–Wallis test, we analysed the Neurological score results. Statistical analysis and plotting of all experimental data were performed using the GraphPad Prism 8.0 software (GraphPad Software, Inc., La Jolla, CA, USA). The results of cerebral infarction percentage and Western Blotting were subjected to statistical analyses by one-way ANOVA (multiple groups) or Bonferroni corrected *t-*test (two groups) using the SPSS 21.0. When the variance was homogeneous, the least significant difference test was selected; otherwise, a non-parametric test was used. Statistical significance was considered at *p < 0.05 and **p < 0.01 for all tests. All data were expressed as the mean ± standard deviation (SD).

Clean data were extracted from the Raw data using USEARCH 8.0. Operational Taxonomic Units (OTUs) were classified based on 97% similarity after chimeric sequences were removed using UPARSE (version 7.1 http://drive5.com/uparse/). The phylogenetic affiliation of each 16S rRNA gene sequence was analysed by RDP Classifier (http://rdp.cme.msu.edu/) against the RDP database (RDP Release 11) using a confidence threshold of 70%. The sample diversity metrics were assessed on the basis of the nonparametric Shannon–Wiener diversity index and Simpson diversity index. The non-parametric Mann–Whitney *U-*test was performed to test the significant differences between the two groups. A comparison of multiple groups was performed using a nonparametric Kruskal–Wallis test. Both weighted UniFrac and unweighted UniFrac were calculated in QIIME, and the QIIME pipeline was used to generate the principal coordinate analysis (PCA) plots so as to visualize the unweighted UniFrac phase differences. Statistical significance between the groups was tested with PERMANOVA using 10,000 permutations (QIIME package). Taxa with differences in the abundance between taxa were analysed by linear discriminant analysis (LDA) effect size (LEfSe). Bar graphs, PCoA plots, subject working characteristic curves, and area under subject working characteristic curves values were generated in the R software (http://www.R-project.org/).

## Results

### HQ-HH improves the neurological deficits induced by MCAO and decreases the infarct volume and the rate of necrotic neurons

**Figure 1.**
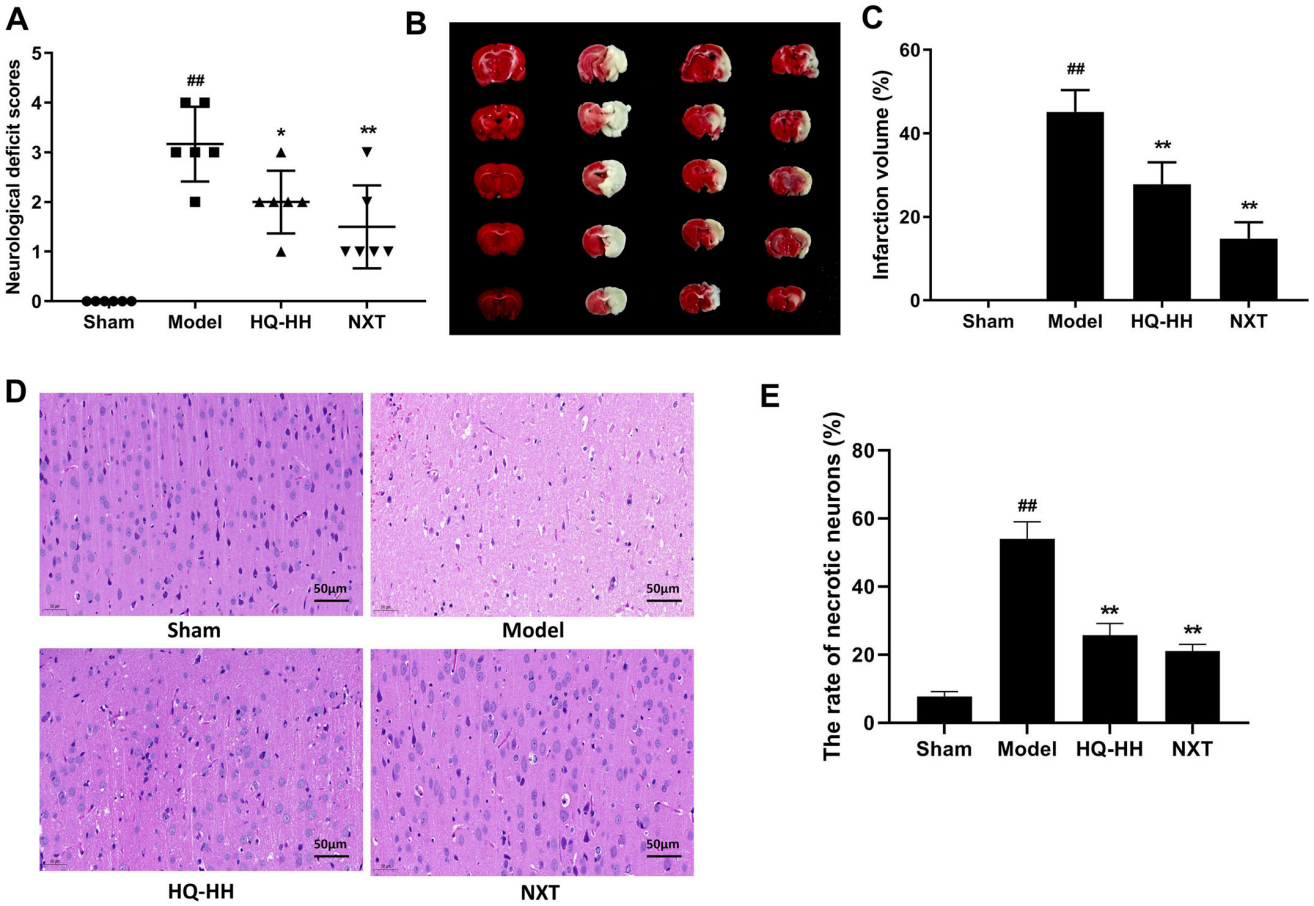
HQ–HH improves the neurological deficits induced by MCAO, reduces the infarct volume, and decreases the rate of necrotic neurons. (A) Scatterplot of the neurological deficits in the Sham, Model, HQ-HH, and NXT groups (data were expressed as median, *n* = 6). (B) The cerebral infarct volume of rats in each group. (C) Statistical analysis of the rate of the infarct volume in each group. (D) H and E staining of the coronal slices of the ischaemic cerebral cortex. (E) Statistical analysis of the rate of necrotic neurons in each group. All data, except for the neurological score, were expressed as the mean ± SD; ^##^*p* < 0.01, when compared with the sham group; *p < 0.05 or **p < 0.01, when compared with the model group.

The neurological scores at 72 h after reperfusion are shown in Figure 1(A). No neurological deficit was observed in the sham group and a severe neurological deficit in the model group (*p* < 0.01 vs. sham group). Compared with the model group, HQ-HH significantly reduced the neurological deficit scores of the model rats (2.0 ± 0.2 vs. 3.16 ± 0.56, *p* < 0.05). Similarly, no infarct volume was detected in the sham group, whereas the model group had a larger infarct volume (*p* < 0.01). Compared with the model group, HQ-HH significantly reduced the cerebral infarct volume (27.83 ± 3.95 vs. 45.17 ± 2.75, *p* < 0.01, Figure 1(B,C)). HE staining further confirmed the neuroprotective effect of HQ-HH on brain injury. As shown in Figure 1(D), neurons in the sham group were regularly arranged with distinct cell outlines and nuclei located in the centre of the cells. The percentages of necrotic neurons in the medullary nuclei of rats in the model group were significantly higher than those in the sham group (*p* < 0.01). Compared with the model group, HQ-HH significantly reduced the rate of necrotic neurons (26.35 ± 4.37 vs. 53.50 ± 9.61, *p* < 0.01).

### HQ-HH altered the composition and function of gut microbiota in rats with CI/RI

**Figure 2.**
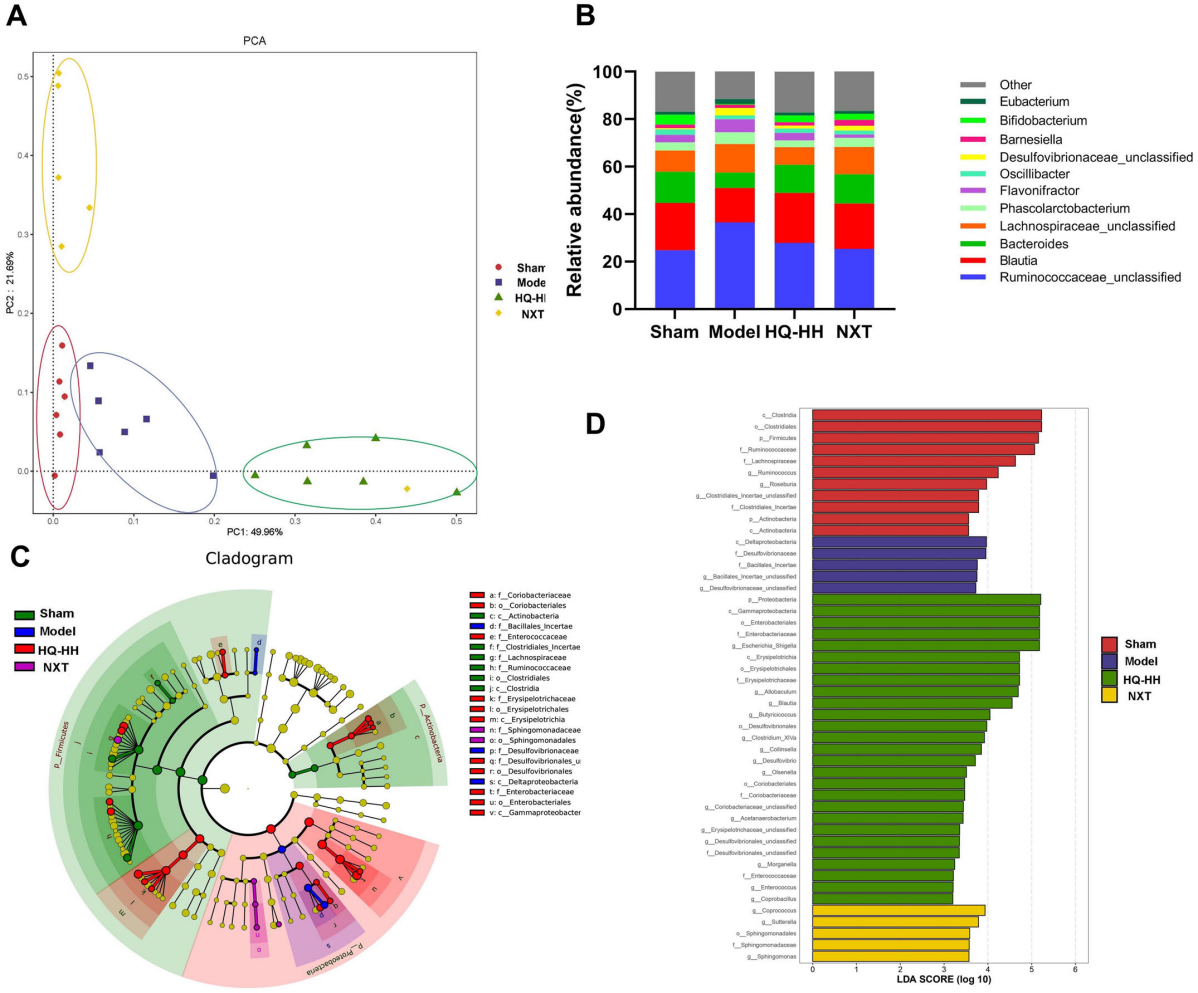
HQ–HH altered the composition and function of gut microbiota in rats with cerebral ischaemia–reperfusion injury. (A) Weighted UniFrac principal coordinate analysis PCA plot based on the operational taxonomic units OTUs abundance of each mouse. Each point in the plot represents the gut microbiota of one rat. (B) The abundance of gut microbiota in each group at the genus level. (C) In the diagram of evolutionary branch, the node size indicates the abundance, and it is arranged outward from the phylum to genus by default. Different colour areas indicate different groups. Creatures without significant difference are coloured in yellow, and the biomarkers are coloured according to the groups. Data were expressed as the mean ± standard deviation (SD) (*n* = 6). Differences were assessed by one-way ANOVA with a *post hoc* Student–Newman–Keuls test (*p < 0.05, **p < 0.01, and ns represents no significant difference). (F) Differences in bacterial abundance were generated from linear discriminate analysis effect size LEfSe analysis.

Presently, 16S rRNA molecular amplification and sequencing technology has become a common analysis method to determine intestinal flora diversity, which can achieve rapid and accurate detection and identification of intestinal flora (Clarridge 2004). To examine whether the anti-CI/RI effect of HQ-HH is associated with gut microbiota, we sequenced the bacterial 16S rRNA V4 region from the faeces. PCA can determine the two coordinate axes that show the difference between samples to the greatest extent so that the difference of multi-dimensional data can be shown on the two-dimensional coordinate graph, thereby revealing the simple laws under the background of complex data. The more similar the community composition of samples, the closer the distance of each sample in the PCA chart. The samples with large population differences were far apart. The beta diversity was assessed by PCA using weighted UniFrac distance matrices (Fouts et al. 2012). The results showed that the gut microflora of each group was located in different regions, which indicated the gut microflora structure was significantly different among each group (Figure 2(A)). The genus level of gut microbiota indicated that HQ-HH and NXT groups significantly decreased the relative abundance of Ruminococcaceae, *Bacteroides*, *Phascolarctobacterium,* and Desulfovibrionaceae, and increased the relative abundance of *Blautia*, Lachnospiraceae, *Oscillibacter,* and *Bifidobacterium*. The results showed that HQ-HH can alter the relative abundance of gut microbiota back to that of the sham group (Figure 2(B)).

LEfSe is a software used to determine high-dimensional biosignatures and reveal genomic features. Its algorithm emphasizes the statistical significance and biological relevance. The histograms of LDA value distributions show organisms with larger settings of LDA scores (default settings), which are biomarkers of statistical differences between the groups (Chen et al. 2015). It shows significant differences in the gene abundance across groups, and the length of the histogram indicated the degree of influence (LDA score) of different organisms (Figure 2(D)). In the diagram of the evolutionary branch, the node size indicates the abundance and is arranged outward from phylum to genus by default, and different colour areas indicate different groups (Gao et al. 2018). The colouring principle is as follows: organisms without significant differences are painted yellow, and biomarkers are coloured by group, with green nodes showing microbial taxa that play an important role in the green group, blue nodes showing microbial groups that play an important role in the blue group, red nodes showing microbial groups that play an important role in the red group, and purple nodes showing microbial groups that play an important role in the purple group (Figure 2(C)). The results of species difference analysis showed that *Clostridia*, *Clostridiales*, *Firmicutes*, had a higher abundance and were the dominant species in the sham group. *Desultaproteobacteri*, Desulfovibrionaceae, and *Bacillales_incertae* were the dominant species in the model group with high abundance. In the HQ–HH group Proteobacteria, Gammaproteobacteri, Enterobacteriales, and Enterobacteriaceae had a higher abundance. The abundance of *Coprococcus*, *Sutterella*, and Sphingomonadales were higher in the NXT group.

### HQ–HH increased intestinal barrier integrity and activated bile acid receptor FXR

**Figure 3.**
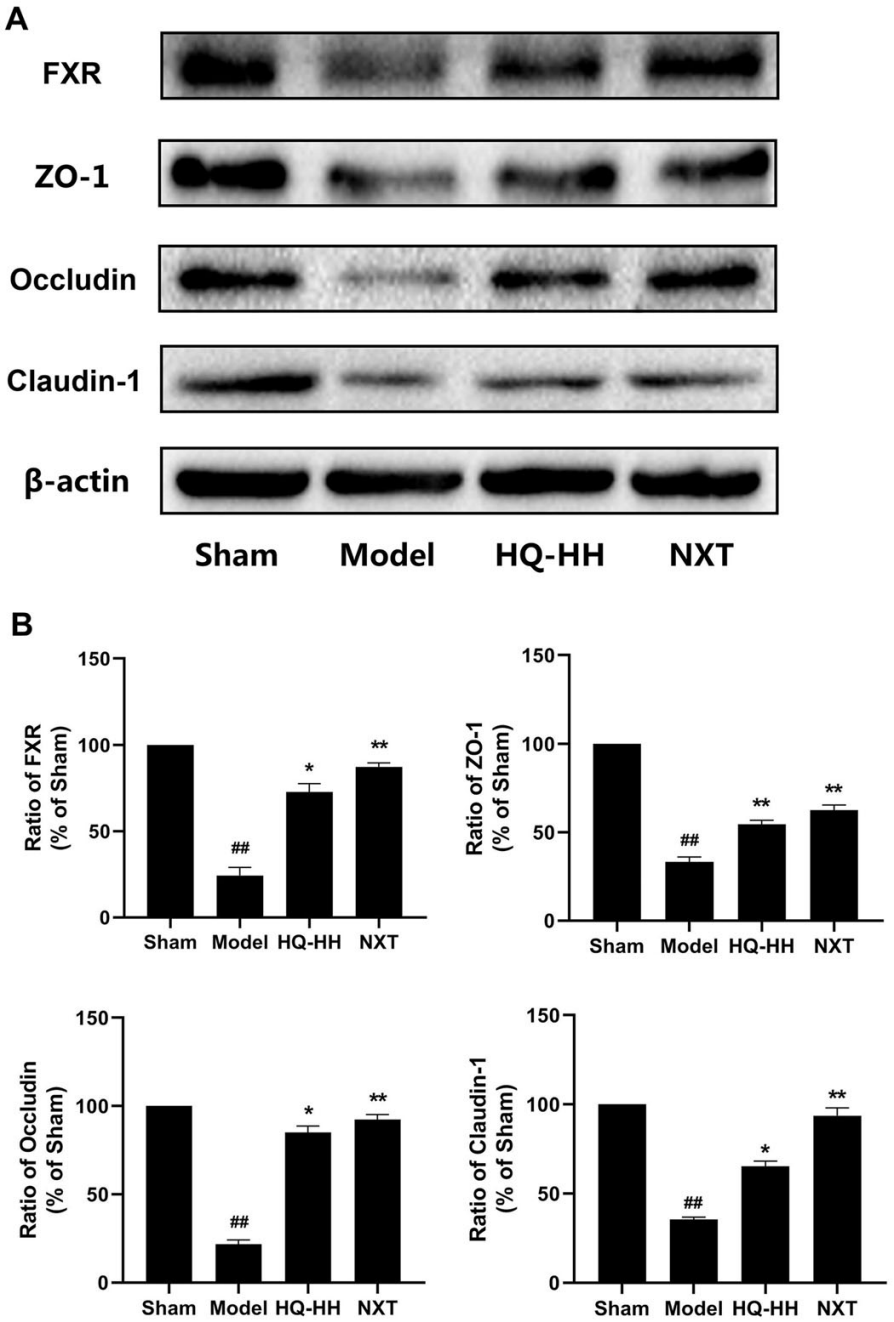
HQ–HH enhanced the intestinal barrier integrity and activated the bile acid receptor FXR. The relative expression of FXR, ZO-1, occludin, and claudin-1 in the small intestine of the Sham, Model, HQ-HH, and NXT groups were detected by Western blotting. (A) Representative bands exhibited the relative expression of FXR, ZO-1, Occludin, and Claudin-1. (B) Statistical analysis results of the detected proteins in each group. Data were presented as the mean ± SD. ^##^*p* < 0.01, compared with the sham group; *p < 0.05 or **p < 0.01, compared with the Model group.

Gut microbiota plays an important role in metabolism, immune function, and maintenance of intestinal barrier function. The homeostasis of gut microbiota is closely associated with human health. The dysbiosis of the intestinal flora can destroy the mucosal barrier and harmful substances can invade. This can lead to various diseases (Roubalová et al. 2020). Epithelial cells are mainly connected by compact proteins, which form the mucosal barrier and maintain the integrity of the barrier function (Daniele et al. 2018). Occludin, *Zonula occludens* 1 (ZO-1), and Claudins play a crucial role in the composition of tight junction proteins (Fermín et al. 2014). FXR is the regulator of bile acid concentration in the liver and intestinal cells. It participates in the regulation of bile acid levels in hepatointestinal circulation and maintains bile acid homeostasis in the body. We evaluated the levels of junction proteins in the tissues of the small intestine to determine whether HQ-HH can attenuate stroke-associated disruption of intestinal architecture. The results showed that the protein levels of ZO-1, occludin, and claudin-1 were significantly decreased in the model group compared with those in the sham group (*p* < 0.01). The disturbance of the gut microbiota after cerebral ischaemia can lead to the destruction of the intestinal barrier. The treatment of cerebral ischaemia rats by HQ-HH can significantly increase the protein levels of ZO-1, occludin, and claudin-1 (*p* < 0.05 or *p* < 0.01; Figure 3(A,B)). We also found that the protein levels of FXR were significantly decreased in the model group than those in the sham group (*p* < 0.01). The treatment of cerebral ischaemia rats with HQ-HH significantly increased the protein levels of FXR (*p* < 0.05; Figure 3(A,B)).

### HQ–HH regulates bile acid metabolism and IL-17A and Foxp3 expression in the small intestine of rats

**Figure 4.**
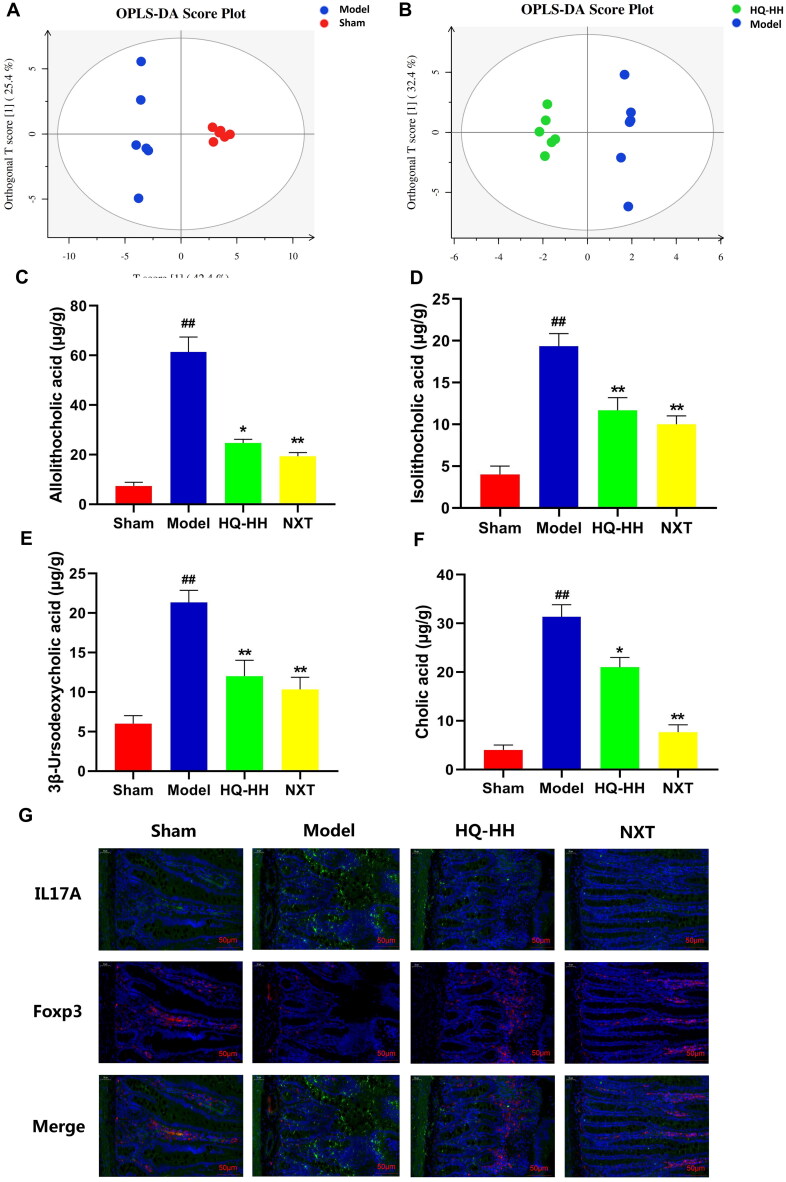
HQ–HH regulates bile acid metabolism and the expression of IL-17A and Foxp3 in the small intestine of rats. The orthogonal partial least-squares discriminant analysis OPLS-DA scores plots discriminated (*n* = 6). (A) Sham and Model group. (B) Model and HQ–HH group. Bile acid including allolithocholic acid (C), isolithocholic acid (D), 3β-ursodeoxycholic acid (E), and cholic acid (F) in the faeces were determined by LC-MS. (G) The expression of IL-17A and Foxp3 in the small intestine was detected by immunofluorescence.

Metabolomics is an excellent tool for revealing crosstalk information between the host and the gut microbiota (Bernstein et al. 2009; Org et al. 2017). In recent years, the application of metabolomics to study the action mechanisms of TCM has developed rapidly and is crucial for the modernization of TCM (Ji et al. 2015). Bile acids play an important regulatory role in various pathophysiological processes such as inflammation, the structure and growth of intestinal flora, and the occurrence and development of diseases such as atherosclerosis, obesity, and cholestasis (Devlin and Fischbach 2015; Chiang and Ferrell 2018). Therefore, we analysed the bile acid using LC/MS (Tautenhahn et al. 2008). In this study, we performed orthogonal partial least square discrimination analysis (OPLS-DA) differently to analyse multidimensional data (Genneback et al. 2013). First, OPLS-DA was performed to analyse the bile acid data of rat faeces samples. The OPLS-DA score plot presented a distinct clustering of the gut metabolite, bile acid, in the faeces samples between the sham and model groups, as well as the model and HQ-HH groups, suggesting that both CI/RI and HQ-HH affected metabolic profiles (Figure 4(A,B)). Then, we identified different metabolites, and a total of 38 bile acids were annotated. Among these 38 bile acids in the faeces samples, four bile acids including allolithocholic acid (AlloLCA), isolithocholic acid (IsoLCA), 3β-ursodeoxycholic acid (Beta-UDCA), and cholic acid (CA) were significantly increased in the model group compared with the sham group, whereas HQ-HH treatment significantly decreased the concentrations of the four bile acids in the CI/RI rats (*p* < 0.05 or *p* < 0.01; Figure 4(C–F)). The immunofluorescence results of the small intestines of the rats are presented in Figure 4(G). Compared with the sham group, IL-17A fluorescence (green) in the small intestine tissues of the model group was enhanced, indicating the increased IL-17A expression in the small intestine tissues, whereas FOXP3 fluorescence (red) in the small intestine tissues was weakened, indicating the decreased FOXP3 expression in the small intestine tissues. Compared with the model group, IL-17A fluorescence in the HQ-HH and NXT groups decreased, indicating the decreased IL-17A expression in the small intestine, whereas Foxp3 fluorescence in the small intestine increased, indicating the increased Foxp3 expression in the small intestine.

### HQ–HH regulates Th17 and Treg cells in the brain of cerebral ischaemia–reperfusion rats, reduces the inflammatory response, and protects the integrity of the blood–brain barrier

**Figure 5.**
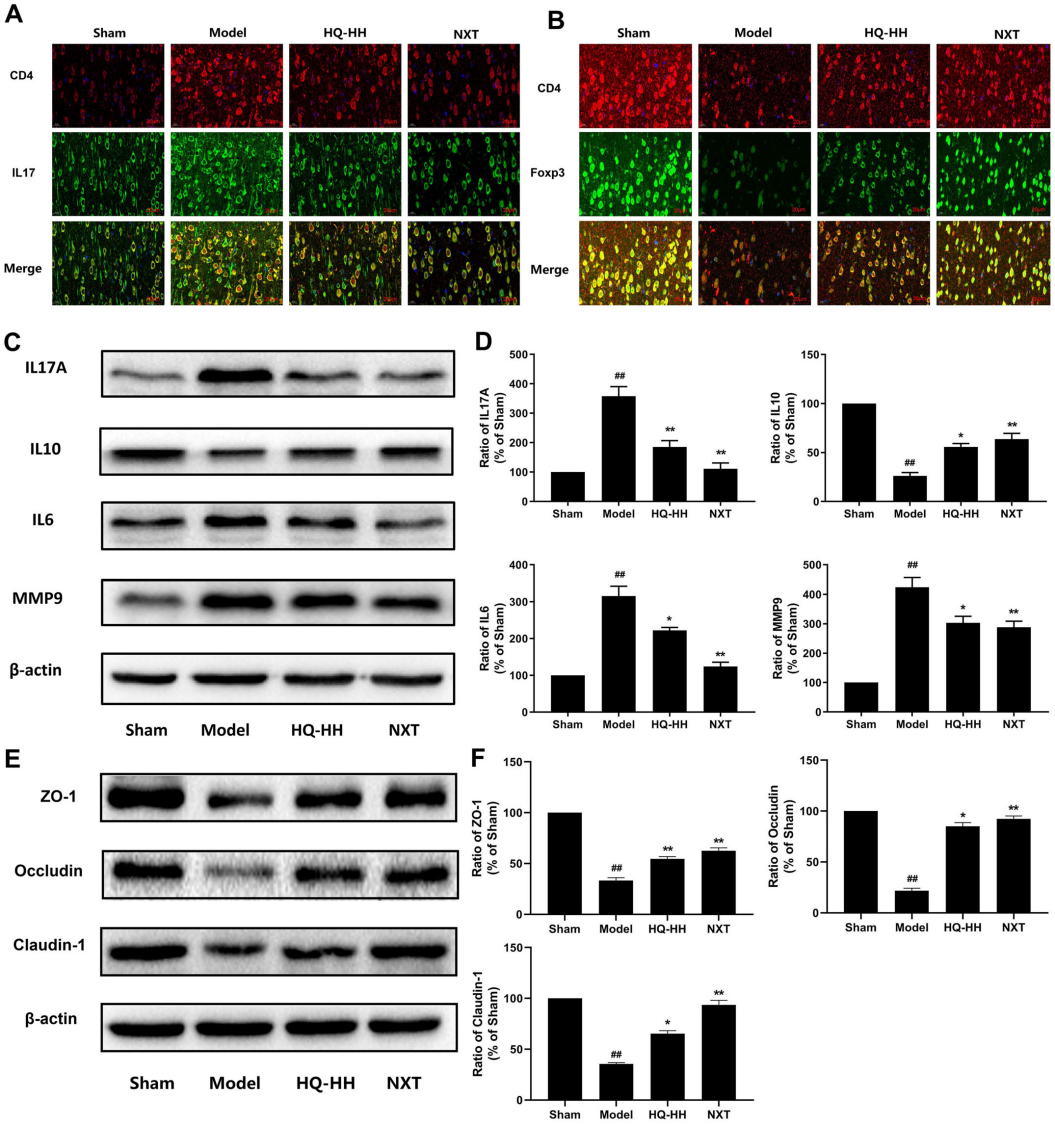
HQ–HH regulates Th17 and Tregs cells in the brain of cerebral ischaemia–reperfusion rats, reduces inflammatory response, and protects the integrity of the blood–brain barrier. (A) The red signal of immunofluorescence indicates CD4^+^ cells, the green signal indicates IL-17^+^ cells, and the yellow area indicates CD4^+^ IL-17^+^ cells (Th17 cells). (B) The red signal of immunofluorescence indicates CD4^+^ cells, the green signal indicates Foxp3^+^ cells, and the yellow area indicates CD4^+^ Foxp3^+^ cells (Tregs cells). The relative expression of IL-17A, IL-10, IL-6, and MMP-9 in the cerebral cortex of the Sham, Model, HQ–HH, and NXT groups detected by Western blotting. (C) The representative bands exhibiting the relative expression of IL-17A, IL-10, IL-6, and MMP-9. (D) Statistical analysis of the proteins detected in each group. The relative expression of ZO-1, occludin, and claudin-1 in the cerebral cortex of the Sham, Model, HQ–HH, and NXT groups detected by Western blotting. (E) Representative bands exhibiting the relative expression of ZO-1, occludin, and claudin-1. (F) Statistical analysis of the proteins detected from each group. Data are presented as the mean ± SD. ^##^*p* < 0.01, when compared with the sham group; *p < 0.05 or **p < 0.01, when compared with the Model group.

The balance between Th17 and Treg cells is vital in the pathophysiological occurrence and development of ischaemic stroke (Li et al. 2013; Dolati et al. 2018). Bile acid metabolites control host immune responses by directly maintaining the balance of TH17 and Treg cells. The immunofluorescence results of brain tissues are presented in Figure 5(A). The red and green immunofluorescence signals corresponded to CD4^+^ and IL-17^+^ cells, and the yellow area corresponded to CD4^+^ IL-17^+^ cells (Th17 cells), which were mainly concentrated in the cerebral cortex. Compared with the sham group, Th17 cells in the brain tissues of the model group increased significantly. Compared with the model group, Th17 cells in the brain tissues of the HQ-HH and NXT groups decreased significantly. As shown in Figure 5(B), the red and green immunofluorescence signals corresponded to CD4^+^ and Foxp3^+^ cells, and the yellow area corresponded to CD4^+^ Foxp3^+^ cells (Tregs cells), which were mainly concentrated in the cerebral cortex. Compared with the sham group, Treg cells in the brain tissues of the model group were significantly reduced. Compared with the model group, Treg cells in the HQ-HH and NXT groups increased significantly. Th17 cells secrete IL-17A. IL-17A can stimulate the expression of tumour necrosis factor-α, IL-6, and other pro-inflammatory factors, chemokines, and adhesion molecules, and mediate the inflammatory response, thus aggravating the damage to the blood–brain barrier and brain tissues. In the acute stage of cerebral infarction, matrix metalloproteinase 9 (MMP-9) level in brain tissues increases, leading to the hydrolysis of the tight junction protein on the basement membrane of cerebral vessels. As shown in Figure 5(C,D), compared with the sham group, IL-17A, IL-6, and MMP-9 levels increased significantly in the model group (*p* < 0.01), whereas IL-10 levels decreased significantly (*p* < 0.01). Compared with the model group, IL-17A, IL-6, and MMP-9 levels decreased significantly in the HQ-HH and NXT groups (*p* < 0.05), whereas IL-10 levels increased significantly (*p* < 0.01). Moreover, as shown in Figure 5(E,F), compared with the sham group, t ZO-1, occludin, and claudin-1 levels decreased significantly in the model group (*p* < 0.01). Compared with the model group, ZO-1, occludin, and claudin-1 levels increased significantly in the HQ-HH and NXT groups (*p* < 0.05). Occludin, ZO-1, and claudin-1 play an important role in the composition of tight junction proteins in the brain. To summarize, HQ-HH regulates the differentiation of Th17 and Treg cells, reduces the levels of pro-inflammatory cytokines IL-17A and related inflammatory mediators, increases the levels of anti-inflammatory cytokines IL-10, and reduces the destruction of the blood–brain barrier and neuroinflammatory response mediated by inflammation after ischaemia.

## Discussion

Ischaemic stroke has high morbidity, disability, and mortality and seriously endangers human life (Ji et al. 2017). CI/RI is a key complication affecting the clinical prognosis of ischaemic stroke. Therefore, exploring drugs to improve CI/RI is of great importance. Several hundred years of practice have confirmed the efficacy of TCM in treating ischaemic stroke. HQ-HH comes from Buyang Huanwu decoction, a famous Chinese medicine prescription for treating ischaemic stroke (Wang et al. 2011). We found that HQ-HH reduced the area of cerebral infarction in rats with cerebral ischaemia–reperfusion, improved damaged nerve function and nerve cell morphology, and exhibited a certain protective effect on CI/RI. Moreover, we also observed that HQ-HH could regulate the gut microbiota, activate the bile acid receptor FXR, maintain the body bile acid homeostasis, decrease Th17 cells and increase Treg cells in the rat brain, decrease the level of the pro-inflammatory factor IL-17A and anti-inflammatory factor IL-10, reduce the inflammatory response in the brain, and protect the integrity of the blood–brain barrier, thereby ameliorating CI/RI in the rats.

The analysis of the faecal microbiota showed that HQ-HH improved gut microbiota dysbiosis in the cerebral ischaemia–reperfusion rats. *Blautia* is a common intestinal beneficial bacterium at the genus level, which produces acetic acid, lactic acid to promote intestinal health and reduce childhood obesity and intestinal inflammation (Benitez-Paez et al. 2020). *Bifidobacterium* is an intestinal probiotic that can inhibit the growth of putrefying and pathogenic bacteria, regulate the intestinal environment, promote intestinal health, and protect the intestinal barrier (Sanz et al. 2007). In this study, we found that *Blautia* and *Bifidobacterium* were decreased in the intestinal tract of the cerebral ischaemia–reperfusion rats, whereas HQ-HH could increase their relative abundance and increase ZO-1, occludin, and claudin-1 levels in the small intestine to protect the intestinal barrier function. Desulfovibrionaceae is a sulphate-reducing bacterium closely related to the development of ulcerative colitis, obesity, insulin. *Bacteroides* are common bacteria in the intestine. When the gut microbiota is stable, it has a mutually beneficial effect on the host; however, when the intestinal barrier function is destroyed and the flora is disturbed, pathological changes such as abscess or bacteraemia can occur (Wexler 2007). We found that HQ-HH could reduce the relative abundance of Desulfovibrionaceae and *Bacteroides* in the intestine of rats with CI/RI, and regulate the homeostasis of the gut microbiota. Therefore, the abnormal increase of Desulfovibrionaceae and *Bacteroides* in the intestine may be a potential mechanism for aggravating CI/RI.

Gut microbiota can regulate the binding and resorption of bile acids via the FXR, thereby affecting the diversity of bile acids (Goodwin et al. 2000). FXR is a member of the superfamily of nuclear receptors, mainly distributed in organs including the liver and small intestine. Bile acid is its endogenous ligand; hence, it is also called the bile acid receptor; it plays a crucial role in bile acid synthesis and metabolism (Ferrebee and Dawson 2015). It also regulates the energy metabolism, lipid metabolism, and immune function of the host (Kim et al. 2007; Bernstein et al. 2009). Interestingly, we found that HQ-HH could activate the expression of the FXR gene in the small intestine of the rats and decrease the contents of AlloLCA, IsoLCA, beta-UDCA, and CA in the faeces of the rats after cerebral ischaemia–reperfusion. Bile acid is an important signalling molecule in the body. Maintaining the homeostasis of bile acid plays a vital role in preventing bile acid toxicity and inflammation in the gastrointestinal tract (Halilbasic et al. 2013; Chiang 2017). Bile acids can regulate immune homeostasis and intestinal barrier function, as well as affect the differentiation of Th17 and Treg immune cells (Hang et al. 2019). The balance of Th17 and Treg cells is crucial in the pathophysiology and development of ischaemic stroke (Li et al. 2013). We found that HQ-HH could decrease IL-17A and increase Foxp3 levels in the small intestines of the rats. However, IL-17 is a key effector factor of Th17 cells, and Foxp3 is one of the key transcription factors that control the development and function of Treg cells and is also a marker molecule of Treg cells; hence, their levels can reflect the differentiation status of Th17 and Treg cells. Interestingly, the immunoassay results in the brain tissues showed that Th17 cells decreased and Treg cells increased in the Astragalus-Safflower group, which was consistent with the levels in the small intestine. IL-17A can stimulate the expression of the genes of various pro-inflammatory factors, chemokines, and adhesion molecules such as IL-6 and mediate inflammatory responses, thereby aggravating the damage to the blood–brain barrier and brain tissues (Shichita et al. 2009; Waisman et al. 2015). In the acute phase of cerebral infarction, the MMP-9 level in the brain tissue increases, leading to the hydrolysis of tight junction proteins on the basement membrane of cerebral blood vessels, thereby destroying the integrity of the blood–brain barrier, which can induce brain edoema and aggravate brain injury (Jonckheere et al. 2011; Rempe et al. 2016). IL-10 is an anti-inflammatory factor secreted by Treg cells, which exerts a neuroprotective effect after ischaemic stroke (Dou et al. 2019). We found that IL-17A, IL-6, and MMP-9 levels in the brain tissues of the HQ-HH group significantly decreased, whereas IL-10 as well as ZO-1, occludin, and claudin-1 levels significantly increased. Therefore, HQ-HH can regulate the differentiation of Th17 and Treg cells in the brain, reduce the levels of the pro-inflammatory factor IL-17A and related inflammatory mediators, increase the level of the anti-inflammatory factor IL-10, and alleviate the inflammatory blood–brain barrier destruction and neuroinflammatory response after ischaemia. This study also provides a new mechanism for the neuroprotective effect of HQ-HH, suggesting that HQ-HH may have a therapeutic value in acute ischaemic stroke. These results provide useful information to better understand the role of HQ-HH in ischaemic stroke and further strengthen the therapeutic value of HQ-HH in ischaemic stroke. However, we only examined the effects of HQ-HH on gut microbiota and bile acid metabolism and did not study the relationship between them. Therefore, we will further explore the effect of HQ-HH on bile acid metabolism after regulating intestinal flora.

## Conclusions

In the present study, we showed the protective effect of HQ-HH on CI/RI in the rat brain. This effect may be achieved by regulating gut microbiota, activating the bile acid receptor FXR, maintaining the homeostasis of bile acid, reducing Th17 cells and increasing Treg cells in the rat brain, reducing the level of the pro-inflammatory cytokine IL-17A, and increasing the level of the anti-inflammatory factor IL-10, reducing the inflammatory response of the brain, protecting the integrity of the blood–brain barrier, and improving CI/RI. The present study provides important experimental evidence for the development of HQ-HH as a potential anti-ischaemic stroke drug, and intestinal flora may be a potential drug-targeting area for HQ-HH in treating ischaemic stroke.
